# Influence of *TFAP2B* and *KCTD15* genetic variability on personality dimensions in anorexia and bulimia nervosa

**DOI:** 10.1002/brb3.784

**Published:** 2017-07-27

**Authors:** Carmen Gamero‐Villarroel, Luz M. González, Raquel Rodríguez‐López, David Albuquerque, Juan A. Carrillo, Angustias García‐Herráiz, Isalud Flores, Guillermo Gervasini

**Affiliations:** ^1^ Department of Medical & Surgical Therapeutics Division of Pharmacology Medical School University of Extremadura Badajoz Spain; ^2^ Service of Clinical Analyses General University Hospital Valencia Spain; ^3^ Research Center for Anthropology and Health (CIAS) University of Coimbra Coimbra Portugal; ^4^ Eating Disorders Unit Institute of Mental Disorders Health Service of Extremadura Badajoz Spain

**Keywords:** anorexia nervosa, body mass index, bulimia nervosa, eating disorders, epistasis, feeding, genetic polymorphisms, KCTD15, obesity, TFAP2B

## Abstract

**Introduction:**

*TFAP2B* and *KCTD15* are obesity‐related genes that interact to regulate feeding behavior. We hypothesize that variability in these loci, isolated or in combination, could also be related to the risk of eating disorders (ED) and/or associated psychological traits.

**Methods:**

We screened 425 participants (169 ED patients, 75 obese subjects, and 181 controls) for 10 clinically relevant and tag single‐nucleotide polymorphisms (SNPs) in *KCTD15* and *TFAP2B* by the Sequenom MassARRAY platform and direct sequencing. Psychometric evaluation was performed with EDI‐2 and SCL‐90R inventories.

**Results:**

The *KCTD15* rs287103 T variant allele was associated with increased risk of bulimia nervosa (BN) (OR = 4.34 [1.47–29.52]; *p* = .003) and with scores of psychopathological scales of these patients. Haplotype *6 in *KCTD15* was more frequent in controls (OR = 0.40 [0.20–0.80], *p* = .009 for anorexia nervosa), while haplotype *4 in *TFAP2B* affected all three scales of the SCL‐90R inventory in BN patients (*p* ≤ .01). Epistasis analyses revealed relevant interactions with body mass index of BN patients (*p* < .001). Genetic profiles in obese patients did not significantly differ from those found in ED patients.

**Conclusions:**

This is the first study that evaluates the combined role of *TFAP2B* and *KCTD15* genes in ED. Our preliminary findings suggest that the interaction of genetic variability in these loci could influence the risk for ED and/or anthropometric and psychological parameters.

## 1 INTRODUCTION

Eating disorders (ED) are complex psychiatric disorders that have become the third most chronic disease among young females in western societies (Herpertz‐Dahlmann, 2009). It is now widely accepted that genetic factors are involved in the etiology of ED and many association studies in this regard have been carried out in the last decade (Clarke, Weiss, & Berrettini, 2012; Hinney & Volckmar, 2013; Rask‐Andersen, Olszewski, Levine, & Schioth, 2010). However, it is striking that the majority of genes involved in the central regulation of food intake, such as those recently evidenced by genome‐wide association studies (GWAS) and follow‐up studies in obese individuals (Waalen, 2014), have not yet been explored in the ED setting. Two such genes are the Transcription Factor AP‐2 Beta (*TFAP2B*) and the Potassium Channel Tetramerization Domain Containing 15 (*KCTD15*).

The transcription factor AP‐2 family encodes critical regulatory factors for neural gene expression and development that also exert a tight regulation of monoaminergic genes (Damberg, 2005). There is a polymorphic region (rs370476693) in the second intron of *TFAP2B* gene consisting of a variable number of [CAAA] repeats of four or five times, with less number of repeats associated with increased gene expression (Ivorra et al., 2011). This polymorphism has been related to different personality dimensions in women and adolescents (Damberg et al., 2000, 2003; Nilsson, Sjoberg, Leppert, Oreland, & Damberg, 2009; Prichard, Jorm, Mackinnon, & Easteal, 2007), including one study in patients with binge‐eating disorder (BED) (Damberg, Garpenstrand, Hallman, & Oreland, 2001).

**Figure 1:**
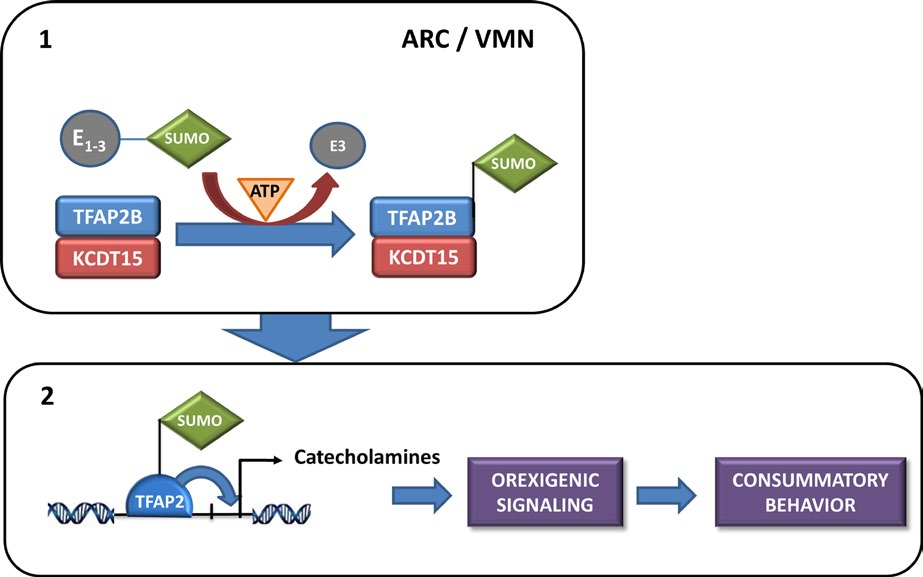
Hypothesis for the involvement of TFAPB and KCTD15 in the regulation of feeding behavior. (1) TFAP2B and KTCD15 co‐localize in Arcuate (ARC) and Ventromedial (VMN) nucleus of the hypothalamus, where KCTD15 would act like a scaffold for the sumoylation of TFAP2B. (2) This post‐translational modification (via E1‐3 enzymes and ATP) would then change TFAP2B function and allow it to induce catecholamines signaling to trigger an orexigenic signal that would eventually lead to consummatory behavior

The *KCTD15* gene is a member of the *KCTD* family with an antagonist role in neural crest formation (Dutta & Dawid, 2010; Zarelli & Dawid, 2013). A number of GWAS have identified SNPs in or near de *KCTD15* gene as putative regulators of body mass index (BMI) (Mei et al., 2012; Paternoster et al., 2011; Thorleifsson et al., 2009; Willer et al., 2009). Interestingly, there is recent evidence in animal models that *TFAP2B* and *KCTD15* homologues co‐localize in areas of the brain where they interact to regulate feeding behavior and reward (Williams et al., 2014) (Figure 1).

Genetic alterations in the complex neural system aimed to keep energy balance by food intake are known to trigger and maintain obesity, but the genes involved in the neuronal control of weight regulation might also be relevant for ED (Day, Ternouth, & Collier, 2009; Gervasini & Gamero‐Villarroel, 2015). Indeed, we and others have shown that variability in these genes is also associated with ED‐related behaviors (Gamero‐Villarroel et al., 2014, 2015; Muller et al., 2012; Rybakowski et al., 2007). According to this background, we hypothesized in the present work that *TFAP2B* and *KCTD15* variants, either isolated or in combination, may influence personality dimensions in anorexia nervosa (AN) or bulimia nervosa (BN) patients. We also investigated whether this effect was different between females with AN and those with BN, who may share more features with obese individuals. A secondary goal was to evaluate differences in genotype and haplotype frequencies between ED patients and both control and obese subjects.

## 2 METHODS

### 2.1 Participants

One‐hundred and sixty‐nine unrelated consecutive female patients with ED (AN = 106, BN = 63), participated in the study. The patients attended the collaborating Eating Disorders Unit at the Mental Health Outpatient Clinic in Badajoz (Spain), and were diagnosed by one psychiatrist and one psychologist using the ED section of the Structured Clinical Interview for Diagnostic and Statistical Manual of Mental Disorders, 4th ed. (Association, 1994). Diagnosis was later re‐evaluated to comply with the new DSM‐V guidelines. Anthropometric (weight, height, and BMI) and psychological parameters (see below) were collected. Diagnosis was blind to genotype. Exclusion criteria, determined upon screening, included dementia, mental retardation, schizophrenia, Turner's syndrome, other neurological disorders, and underlying endocrine pathologies. A total of 181 healthy women from the same geographical area as the patients (Health District of Badajoz, Spain) were also recruited. Interviews were carried out to guarantee that they had never been diagnosed as having any psychiatric disorder or received any psychiatric treatment. All these individuals showed normal BMI values and had no history of ED. Finally, a group of 75 female obese patients with no ED, recruited among patients of the Infanta Cristina University Hospital in Badajoz (Spain) was included for comparison purposes. These subjects were selected from families from the same region as the ED patients that were studied to detect cases of morbid obesity. Obese individuals included reached a weight >3 standard deviations above the mean before 14 years of age and referred at least two other morbid obesity cases among first or second degree relatives. Grade 3 overweight (morbid obesity) was considered to be BMI ≥ 40. Individuals with signs and/or symptoms of syndromic obesity were excluded from the study.

All the participants were white Spanish individuals who gave written informed consent. The study protocol was approved by the Ethics Committee of the University of Extremadura and was conducted in accordance with the Declaration of Helsinki and its subsequent revisions.

### 2.2 SNPs selection

European population (CEU) single‐nucleotide polymorphism (SNP) data were downloaded from the 1000 Genome website (https://www.ncbi.nlm.nih.gov/variation/tools/1000genomes/). We analyzed the coding sequence and adjacent 3′‐ and 5′‐untranslated regions of the *TFAP2B* and *KCTD15* genes (ref. NC_000006.12, NC_000019.10) and tag SNPs were assigned using Haploview v4.2 software (Daly Lab at the Broad Institute, Cambridge, MA, USA; Table 1). The minor allele frequency considered was 10%, and pair‐wise tagging with a maximum *r* ^2^ of .80 was applied to capture common variations. Thus, a set of four tag SNPs was selected for *TFAP2B* and five tag SNPs for *KCTD15*. These SNPs highly correlated with the other variants inside the same linkage disequilibrium block (*r* ^2^ values ranged from .52 to 1.0) and captured 100% of the variability of both genes. All the SNPs were intronic and located within an 18,595‐kb region (chromosome positions 50828921–50847516) and 12,882‐kb region (chromosome positions 33799262–33812144) encompassing the *TFAP2B* gene and *KCTD15* gene, respectively.

**Table 1:** Selected single‐nucleotide polymorphisms in the regions encompassing the *TFAP2B* and *KCTD15* genes

Polymorphism	Position^a^	Region	Alelles	MAF
*TFAP2B*				
rs760900	6:50828921	Intronic	C/T	0.432
rs2817420	6:50845619	3′‐UTR	C/T	0.179
rs7769978	6:50847516	3′‐UTR	C/T	0.105
rs552393576	6:50823879	Intronic	A/G	0.192
rs370476693	6:50823893‐6	Intronic	[CAAA]/‐	0.476
*KCTD15*				
rs736239	19:33799262	Intronic	G/C	0.412
rs287103	19:33801816	Intronic	C/T	0.335
rs4239577	19:33803962	Intronic	C/T	0.319
rs4805059	19:33803996	Intronic	G/A	0.483
rs2056180	19:33812144	3′‐UTR	T/C	0.097

MAF, minor allele frequency; UTR, untranslated region.

a Reference assembly GRCh38.

Because of existing studies reporting an involvement of the *TFAP2B* [CAAA]^4‐5^ SNP (rs370476693) with several psychiatric disorders in women, we also searched for this SNP in the ED patients, as well as for rs552393576 (an A‐to‐G change in the third position of the first of these [CAAA] repeats).

### 2.3 Genotype analysis

Blood samples from all participants were collected and stored at −80°C until analysis. Genomic DNA was isolated from peripheral blood leukocytes in 2‐ml aliquots of whole‐blood samples with a Qiagen blood midi kit (Qiagen, Chatsworth, CA, USA). The purified DNA samples were then stored at 4°C in sterile plastic vials. Genotype analyses for SNPs determination were performed with the single‐base extension polymerase chain reaction Sequenom iPLEX‐Gold and the mass spectrometry‐based platform MassARRAY MALDI‐TOF (Sequenom, San Diego, CA, USA) at the Spanish Genotyping National Centre (CEGEN‐ISCIII). In brief, the analyses consisted of an initial locus specific PCR, followed by single‐base extension using mass‐modified dideoxynucleotide terminators of an oligonucleotide primer, which anneals immediately upstream of the polymorphic site of interest. Using matrix‐assisted laser desorption ionization time‐of‐flight mass spectrometry, the distinct mass of the extended primer identifies the allele (Gabriel, Ziaugra, & Tabbaa, 2009). The [CAAA]^4‐5^ and rs552393576 polymorphisms in the *TAFP2B* gene were genotyped by direct sequencing in the same reaction. Primers and PCR conditions are available upon request.

### 2.4 Study of psychological traits

ED patients were evaluated on the first visit to the collaborating Eating Disorders Unit by experienced clinicians. The patients completed the Eating Disorders Inventory Test 2 (EDI‐2) and the Symptom Checklist 90 Revised self‐reported questionnaires. EDI‐2 was designed to assess ED‐related cognitive and behavioral characteristics by initially measuring eight main subscales (Drive for Thinness, Bulimia, Body Dissatisfaction, Ineffectiveness, Perfectionism, Interpersonal Distrust, Interoceptive Awareness, and Maturity Fears), to which other three (Asceticism, Impulse Regulation, and Social Insecurity) were added in a second version (Garner, 1991). The EDI‐2 test has been validated in the Spanish population showing high internal consistency between the different subscales (Guimera & Torrubia, 1987). The Symptom Checklist 90 Revised inventory, which has also been validated in Spaniards (Derogaitis, 2002), assesses a broad range of psychopathological symptoms. Three general indexes (Global Severity Index, GSI; Positive Symptom Distress Index, PSDI; and Positive Symptom Total, PST) are obtained upon completion of the questionnaire (Derogaitis, 1977).

### 2.5 Statistical analyses

Differences of quantitative variables between groups of study were assessed with the Student's *t*‐test. Single‐marker analyses were carried out using logistic regression models adjusted for age using the *SNPassoc* R package (Gonzalez et al., 2007). Statistical significance threshold after correcting for the 10 SNPs assayed was set at 0.005. For the multiple‐marker analysis, the *p*‐value was adjusted by the number of haplotypes identified in the population. The number of psychopathological scales analyzed was not included in the adjustment, as previous studies in ED patients have claimed this would be too stringent to detect moderate associations with different endophenotypes (Gamero‐Villarroel et al., 2014, 2015; Mercader et al., 2007). Haplotype associations were performed with the *haplo.stats* package, also in the R environment. Gene–gene interaction (epistasis) analyses were performed using log‐likelihood ratio tests adjusted by age (*SNPassoc* package) in a codominant model. In the resulting plots, the diagonal line contains the *p*‐values from likelihood ratio test for the crude effect of each SNP, which are sorted by their genomic position. The upper triangle in the matrix contains the *p*‐values for the interaction (epistasis) log‐likelihood ratio test. Finally, the lower triangle contains the *p*‐values from likelihood ratio test comparing the two‐SNP additive likelihood to the best of the single‐SNP models.

## 3 RESULTS

Clinical and descriptive characteristics of healthy subjects and obese and ED patients are summarized in Table 2. Control subjects displayed significantly higher weight and BMI than AN patients (*p* < .05). Likewise, obese individuals had higher weight and BMI than all the other groups, including BN patients (*p* < 10^−5^). In addition, BN patients scored significantly higher than AN patients in the different scales measured in the psychometric evaluation (Table 2).

**Table 2:** Descriptive and clinical variables of patients with anorexia nervosa (AN) or bulimia nervosa (BN), obesity, and healthy controls

	AN	BN	Obese	Controls
Age, years	18.9 ± 6.2	20.9 ± 8.1	35.23 ± 14.11	33.09 ± 18.12
Height, m	1.60 ± 0.07	1.61 ± 0.06	1.61 ± 7.90	1.61 ± 0.07
Weight, kg	44.28 ± 6.08	59.16 ± 13.65	103.11 ± 17.65***	60.3 ± 10.12*
BMI, kg/m^2^	17.19 ± 2.01	22.71 ± 5.03	39.63 ± 6.27***	22.06 ± 5.17*
Age at onset, yrs	16.9 ± 4.8	17.8 ± 4.9		
EDI‐2	88.84 ± 45.53	117.27 ± 39.14**		
GSI (SCL‐90R)	1.62 ± 0.85	1.96 ± 0.82§		
PST (SCL‐90R)	63.33 ± 21.91	71.11 ± 17.07§		
PSDI (SCL‐90R)	2.18 ± 0.64	2.4 ± 0.62§		

Mean ± standard deviation values are shown.

BMI, body mass index; GSI, Global Severity Index; PSDI, Positive Symptom Distress Index; PST, Positive Symptom Total.

*p < .05 for the comparison between controls and AN patients.

**p < .001 and ^§^ *p* < .05 for the comparison between AN and BN patients.

***p < 10^−5^ for the comparison between obese and the other patients groups.

### 3.1 Single‐marker analyses

Minor allelic frequencies for the polymorphisms assayed are shown in Table 1. Note that the *TFAP2B* rs2272903 polymorphism did not survive quality‐control with the genotyping methods utilized and hence it could not be analyzed. The rs287103 T variant allele in the *KCTD15* gene was significantly associated with increased risk of BN, as it was carried by 63.5% of patients but only 52.9% of controls (OR for carriers vs. non carriers = 4.34 [1.47–29.52]; *p* = .003). The percentage of variability explained by the regression model containing rs287103 and age was 43.3%. Interestingly, the same rs287103 SNP was observed to affect two psychological scales of BN patients, namely *Interoceptive awareness* in the EDI‐2 inventory and the global GSI index in the SCL‐90R questionnaire, although statistical significance did not survive correction by multiple testing (Table 3). In the AN patients, rs552393576 and rs2817420 in *TFAP2B* and rs4805059 and rs4239577 in *KCTD15* showed relevant associations with the scores of several dimensions (Table 4). Also in the 106 AN patients, the rs760900 T variant in *TFAP2B* was found to correlate with BMI [mean BMI (standard error) for CC vs. CT+TT carriers = 17.87 (0.33) and 16.81 (0.24); *p* < .01; *r*‐square value for the influence of the regression model containing rs760900 and age on BMI variability = 0.114]. However, after correction by multiple testing, the level of association decreased to a statistical trend.

**Table 3:** Relevant effects of *TFAP2B* and *KCTD15* SNPs on personality dimensions scores obtained for patients with Bulimia Nervosa

SNP	%	Mean diff. (CI)	*p*
*BD*			
rs7769978			
T/T	79.0	Ref.	.038
C/T	21.0	−5.1 (‐9.7 to −0.4)	
*IA*			
rs287103			
C/C	37.1	Ref.	.039
C/T	50.0	3.83 (0.4–7.3)	
T/T	12.9	−1.34 (−6.5 to 3.8)	
*GSI*			
rs287103			
C/C	36.5	Ref.	.046
C/T	49.2	0.53 (0.09–0.9)	
T/T	14.3	0.04 (−0.6 to 0.7)	
*PSDI*			
rs7769978			
T/T	79.0	Ref.	.016
C/T	21.0	−0.47 (−0.8 to −0.1)	

Percentage of carriers for each genotype and mean differences with 95% confidence intervals (CI) with respect to the wild‐type genotype are shown.

BD, body dissatisfaction; IA, interoceptive awareness; GSI, global severity index; PSDI, positive symptom distress index; SNP, single‐nucleotide polymorphisms.

**Table 4:** Relevant effects of *TFAP2B* and *KCTD15* SNPs on personality dimensions scores obtained for patients with anorexia nervosa

SNP	%	Mean diff. (CI)	*p*
*DT*			
rs552393576			
AA	65.7	Ref.	.004
AG	24.2	−3.82 (−6.7 to −0.9)	
GG	10.1	3.38 (−0.7 to 7.5)	
rs4805059			
GG	29.3	Ref.	.005
AG	56.6	1.86 (−0.8 to 4.6)	
AA	14.1	6.65 (2.7–10.6)	
*BD*			
rs2817420			
CC	63.3	Ref.	.010
CT	33.7	−2.19 (‐5.6 to 1.2)	
TT	3.1	12.7 (3.2–21.7)	
*I*			
rs4239577			
C/C	48.0	Ref.	.006
C/T	44.9	4.34 (1.2–7.4)	
T/T	7.1	−3.14 (−9.2 to 2.9)	
*A*			
rs4239577			
C/C	48.0	Ref.	.004
C/T	44.9	3.44 (1.4–5.5)	
T/T	7.1	−0.14 (−3.8 to 3.6)	
*SI*			
rs552393576			
A/A	65.7	Ref.	.031
A/G	24.2	2.6 (0.1–5.1)	
G/G	10.1	3.5 (0.0–7.1)	
*EDI‐2*			
rs4239577			
C/C	48.0	Ref.	.033
C/T	44.9	23.64 (5.4–41.8)	

Percentage of carriers for each genotype and mean differences with 95% confidence intervals (CI) with respect to the wild‐type genotype are shown.

DT, drive for thinness; BD, body dissatisfaction; I, ineffectiveness; A, ascetism; SI, social insecurity; EDI‐2, total score obtained in the test; SNP, single‐nucleotide polymorphisms.

### 3.2 Multiple‐marker analyses

The frequencies of haplotypes identified in the different groups of study and linkage disequilibrium information are shown in Supplementary Table S1 and Supplementary Figure S1, respectively. Not all the possible allele combinations were found in the population. Most notably, haplotype *6 in *KCTD15* (GCTAC) was far more frequent in controls than in any other study group (OR = 0.42 [0.19–0.94], *p* = .03 for the comparison with BN and OR = 0.40 [0.20–0.80], *p* = .009 for the comparison with AN). Only this last association retained significance after controlling by the number of haplotypes identified. Haplotypes in *TFAP2B* (based only in the three tag SNPs, as controls could not be genotyped for rs370476693 or rs552393576) did not modify the risk for ED (data not shown).

With regard to the impact on the psychometric evaluation, haplotype *4 (CCC[CAAA]^4^A) in *TFAP2B* strongly affected the scores of all three scales of the SCL‐90R inventory in BN patients. Regression analyses adjusted by age revealed that this haplotype combination correlated with lower GSI, PST, and PSDI scores in these women. After correcting for the number of haplotypes, *p*‐values retained significance in the GSI and PSDI scales (Table 5). The effect of the *KCTD15* haplotypes was more diffuse and associations did not survive Bonferroni correction of the data. Haplotype *2 was related to *Drive for thinness* (mean difference with 95% confidence intervals [CI] = 3.25 [0.73–5.76], *p* = .011] in AN patients. In the BN group, two haplotypes (*3 and *4) showed a trend toward lower scores in the *Maturity fears* scale (−3.93 [−6.89 to −0.96], *p* = .012) and (−3.62 [−7.08 to −0.15], *p* = .04), respectively.

**Table 5:** Impact of *TFAP2B* haplotypes on the scores of the SCL‐90R inventory in patients with bulimia nervosa

Haplotype	GSI			PSDI			PST		
	Mean diff	95% CI	*p*‐value	Mean diff	95% CI	*p*‐value	Mean diff	95% CI	*p*‐value
*1	2.24	Ref.	—	2.58	Ref.	—	77.16	Ref.	—
*2	−0.08	(−0.58 to 0.41)	.736	0.01	(−0.03 to 0.04)	.9501	−2.81	(−13.29 to 7.67)	.599
*3	−0.1	(−0.64 to 0.44)	.711	−0.08	(−0.74 to 0.31)	.695	0.7	(−10.72 to 12.12)	.904
*4	−0.50	(−0.82 to −0.18)	.002	−0.36	(−0.61 to −0.12)	.003	−10.15	(−17.88 to −2.42)	.010
*5	−0.23	(−0.77 to −0.31)	.401	−0.19	(−0.58 to 0.21)	.353	−1.74	(−13.19 to 9.71)	.766

GSI, global symptoms index; PSDI; Positive Symptom Distress Index; PST, Positive Symptom Total; Mean diff, mean difference; CI, confidence intervals; Ref., reference haplotype.

### 3.3 Gene–gene interactions

**Figure 2:**
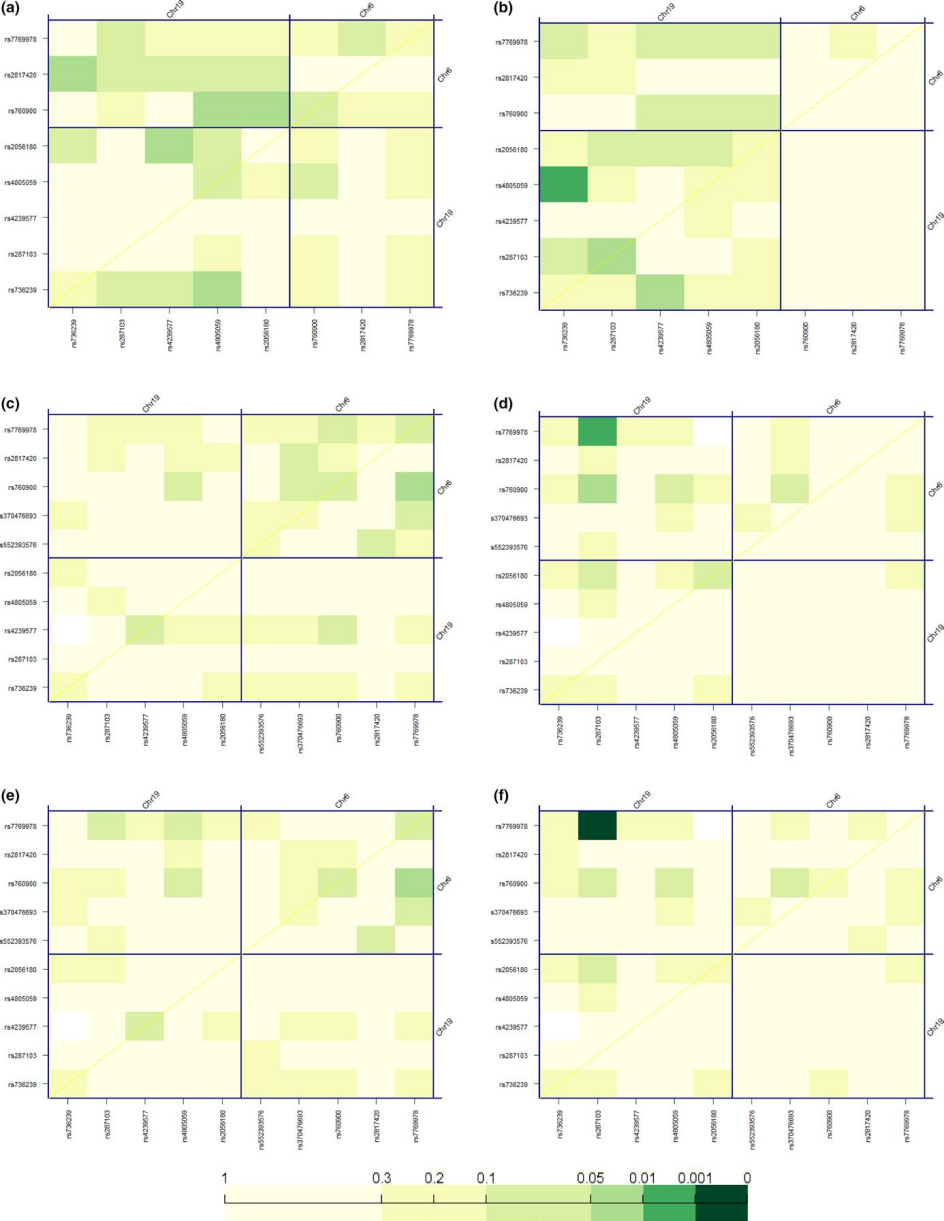
Effect of *TFAP2B*–*KCTD15* interaction on (a) the risk for AN; (b) the risk for bulimia nervosa (BN); (c) weight in AN patients; (d), weight in BN patients; (e), body mass index (BMI) in AN patients and (f), BMI in BM patients. The diagonal line contains the *p*‐values from likelihood ratio test for the crude effect of each single‐nucleotide polymorphisms (SNP), which are sorted by their genomic position. The upper triangle in the matrix contains the *p*‐values for the interaction (epistasis) log‐likelihood ratio test. Finally, the lower triangle contains the *p*‐values from likelihood ratio test comparing the two‐SNP additive likelihood to the best of the single‐SNP models. Chr, chromosome

Figures 2a,b show interactions between genetic variability in *TFAP2B* and *KCTD15* with regard to their effect on the risk for AN and BN adjusted by age. The rs4805059/rs736239 pair showed an association (*p* < .01) with BN risk. In addition, a correlation was observed for the rs776978/rs281703 pair with weight but especially with BMI in these BN patients (*p* < .01 and *p* < .001, respectively; Figure 2d,f).

Finally, we also assessed how interactions between the 10 considered SNPs might affect personality dimensions and psychopathological symptoms in ED patients. The results are depicted in Supplementary Figures S2 and S3. Besides several significant interactions identified for particular SNP pairs (see Figures), a different pattern of interaction was observed in general for AN and BN. In general, the effect of epistasis was more profound in scales such as *Perfectionism*,* Maturity fears* or *Social Insecurity* for AN patients, while in BN patients, dimensions such as *Interpersonal distrust* or *Social insecurity* were found to show more hits.

### 3.4 Obese patients

Out of the eight SNPs assessed in the obesity and control groups, two were associated with obesity risk. The rs2056180 C allele of *KCTD15* was more frequent in controls (26.4% of carriers) than in obese subjects (12.0%) (OR = 0.38 [0.18–0.82]; *p* = .008). In contrast, the *TFAP2B* rs2817420 T allele was present in 29.9% of controls but 45.3% of obese individuals (OR = 1.94 [1.11–3.39], *p* = .02). In any case, the *p*‐values were higher than the threshold established for statistical significance (0.001). Interestingly, this last SNP, rs2817420, was the only one that was differently distributed between ED patients and obese individuals (Supplementary Table S2). As it occurred in BN and AN patients, haplotype *6 in *KCTD15* was significantly less frequent in the obese group than in controls (Supplementary Table S1).

## 4 DISCUSSION

There are many shared personality dimensions and risk factors between obesity and ED. These include body dissatisfaction, low self‐esteem, anxiety, depression, substance abuse, dieting, binge‐eating, and a history of sexual/physical abuse (Day et al., 2009). While some of these traits are the obvious result of the disease, it is likely that the direction of causality runs both ways. Indeed, psychopathological symptoms have been shown to alter eating behavior which in turn leads to obesity (Lee, 2007). Two genes recently associated with obesity have been *TFAP2B* and *KCTD15*, for which a combined function that affects eating behavior has been described (Williams et al., 2014; Zarelli & Dawid, 2013). Following the rationale that argues for a connection between ED and obesity, we could expect that genetic alterations in central pathways involved in the modulation of eating behavior and food intake that may lead to obesity, could also be linked to ED.

Our results showed that the rs287103 T variant allele in the *KCTD15* gene was not only associated with increased risk of BN, but it was also found to modulate psychological features in these women. Furthermore, haplotype *6, which was observed to decrease the risk for ED, contained the “*protecting*” rs287103 C major allele. In contrast, no associations were found for AN risk, which is consistent with the findings by Brandys et al. (2010), who failed to find a significant effect of a *KCTD15* polymorphism previously associated with obesity in AN patients; as well as with several GWAS, which have not identified any *KCTD15* SNP related to AN risk (Boraska et al., 2014; Hinney et al., 2017; Wang et al., 2011). In this regard, several loci in or near *KCTD15* have repeatedly been associated with obesity in a number of GWAS and replication studies (Mei et al., 2012; Paternoster et al., 2011; Thorleifsson et al., 2009; Willer et al., 2009). The cerebral location of the gene indicates that its association with weight increase would likely be based on changes in feeding behavior. Indeed, a recent study has confirmed an association of *KCTD15* SNPs and dietary behaviors in children (Lv et al., 2015) and another report has related variant transcripts of *KCTD15* to fatness traits in animals (Liang et al., 2015). According to our findings, it seems plausible that SNPs in this gene could also help develop aberrant conduct patterns that favor the establishment of ED, should other susceptibility factors or personality dimensions were present. Of note, both SNPs and haplotypes in *KCTD15* were associated in our sample with a number of traits that often appear coupled with ED.

With regard to *TFAP2B,* SNP rs760900 was found to correlate with lower BMI in AN patients (it should be noted, however, that the association lost significance after correction for multiple testing), while other gene variants affected personality dimensions of the ED patients. In addition, haplotype *4 in *TFAP2B* significantly reduced all scores of BN patients in the SCL‐90R inventory. This inventory measures general psychopathological symptoms that do not necessarily have to be intimately related to ED and, as such, would be more typically linked to BN than AN patients. Some of the mechanisms underlying the different behaviors displayed by patients with psychiatric disorders are mediated by the contribution of genes to psychological traits (Cloninger, 1999; Ebstein, Benjamin, & Belmaker, 2000). *TFAP2B* gene variants have previously been related to different personality dimensions in women, namely anxiety, psychasthenia, guilt, depression, impulsiveness, indirect aggression or addictive behavior (Damberg et al., 2000, 2003; Hensch et al., 2008; Nilsson et al., 2009; Nordquist et al., 2009); features that are often present in women with ED. Furthermore, a recent study has also observed an association between *TFAP2B* SNPs and obesity‐related traits (Albuquerque, Nobrega, Rodriguez‐Lopez, & Manco, 2014). Therefore, a link between variability in this gene and ED is also plausible. To the best of our knowledge, there is only one report in the ED setting that addresses this question, although it only analyzed one polymorphism. The authors showed a higher frequency of the [CAAA]^5^ long allele in women with binge‐eating disorders than in controls (Damberg et al., 2001). We could not confirm an important role of the [CAAA]^4‐5^ SNP (rs370476693) in the modulation of psychopathological symptoms in ED patients; however, we did observe that rs552393576, an A‐to‐G transition located within the first repeat of this polymorphism, was indeed associated with certain traits. Consequently, it is tempting to speculate that some of the associations reported for the [CAAA]^4‐5^ polymorphism (Damberg et al., 2000) could be due to rs552393576, which was not searched for in any of the aforementioned clinical studies. But, what would be the precise mechanism linking this gene to these behaviors and specifically to ED? These personality features are expressed by the involvement of a pattern of neuronal adaptations induced by central genes such as dopamine or serotonin, which has been consistently associated with ED‐coupled personality dimensions (Gervasini & Gamero‐Villarroel, 2015). Dopaminergic and serotonergic genes in the midbrain have AP‐2 binding sites; therefore it is likely that these transcription factors, aside from its established role during development, can also influence mood and personality in adults through this neurotransmitter system (Damberg, 2005; Damberg et al., 2001). In the same manner, variability in the *KCTD15* locus could play its role by altering TFAP2B post‐translational modifications leading to orexigenic signaling and consummatory behavior, as shown in Figure 1.

The analysis of obese patients in our sample could confirm that *KCTD15* and *TFAP2B* variants may be related to obesity risk, a finding that has also been suggested by a number of GWAS and replication studies (Albuquerque et al., 2014; Bauer et al., 2009; Lv et al., 2015; Willer et al., 2009). Moreover, the genetic profile of AN, BN and obese patients did not differ significantly, only rs2817420 was differently distributed between the groups. A fact that stresses this similarity in *TFAP2B* and *KCTD15* genetic variability is that the frequency of haplotype *6 in *KCTD15* was significantly higher in controls but was similar between obese and ED patients.

An interesting finding of the present work was that the interaction between genetic variability in *TFAP2B* and *KCTD15* was linked to BN risk as well as correlate with weight and BMI in these patients. These results support previous reports suggesting a combined function of this two genes (Zarelli & Dawid, 2013), and their alleged implication in the regulation of feeding behavior (Williams et al., 2014). It is of note that, overall, the impact of these obesity‐related genes was more evident in BN patients than in the AN group, which makes sense if we bear in mind that alterations in the CNS resulting in behaviors leading to obesity (e.g., anxiety, binge‐eating, etc.) could also lead to bulimia should additional personality traits were present. Their connection with AN would however be more intricate.

Several limitations have to be considered in this study. First, the relatively small size of the population studied might affect the generalizability of these results. However, the fact that the study was performed in the only primary care center specialized in ED in the area, allowed all the patients to be diagnosed and followed in the same facility by the same clinicians, which reduced the chance that the findings may be due to population structure. Second, we could not determine one of the tag *TAFP2B* SNPs and therefore the relevance of the region tagged by this variant could not be assessed. In addition, some of the reported associations for individual SNPs were based on the heterozygous genotype, which probably decrease their clinical significance. Finally, it would have been informative to have EDI‐2 and SCL‐90R scores for the control population, as well as data on *TFAP2B* 5‐SNP haplotypes, which were unfortunately unavailable.

This is the first study that evaluates the combined role of *TFAP2B* and *KCTD15* genes in ED. Our findings, preliminary as they are, given the described limitations, suggest that the interaction of genetic variability in these loci could influence the risk for ED and/or be associated with anthropometric and psychological parameters in these patients. Further studies with larger and homogeneous populations of patients are nevertheless warranted to confirm the reported associations.

## CONFLICT OF INTERESTS

The authors report no conflicts of interest.
