# Supplementary figures and images for: Influence of TFAP2B and KCTD15 genetic variability on personality dimensions in anorexia and bulimia nervosa
Source: Brain Behav. 2017 Jul 27;7(9):e00784. doi: 10.1002/brb3.784 (PMC5607548; doi:10.1002/brb3.784)

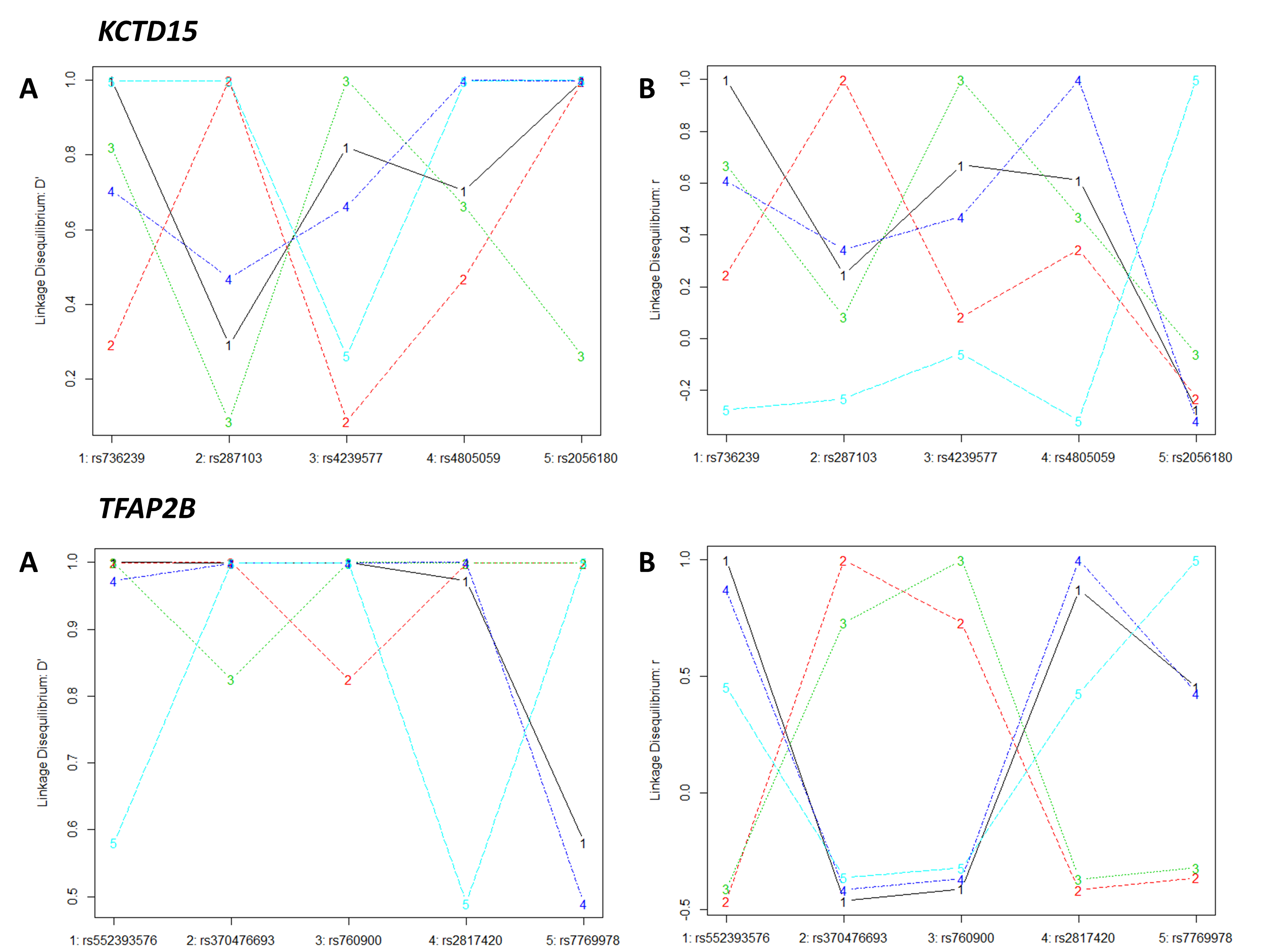

Supplement: Supplementary file 1 [file BRB3-7-e00784-s001.TIF]

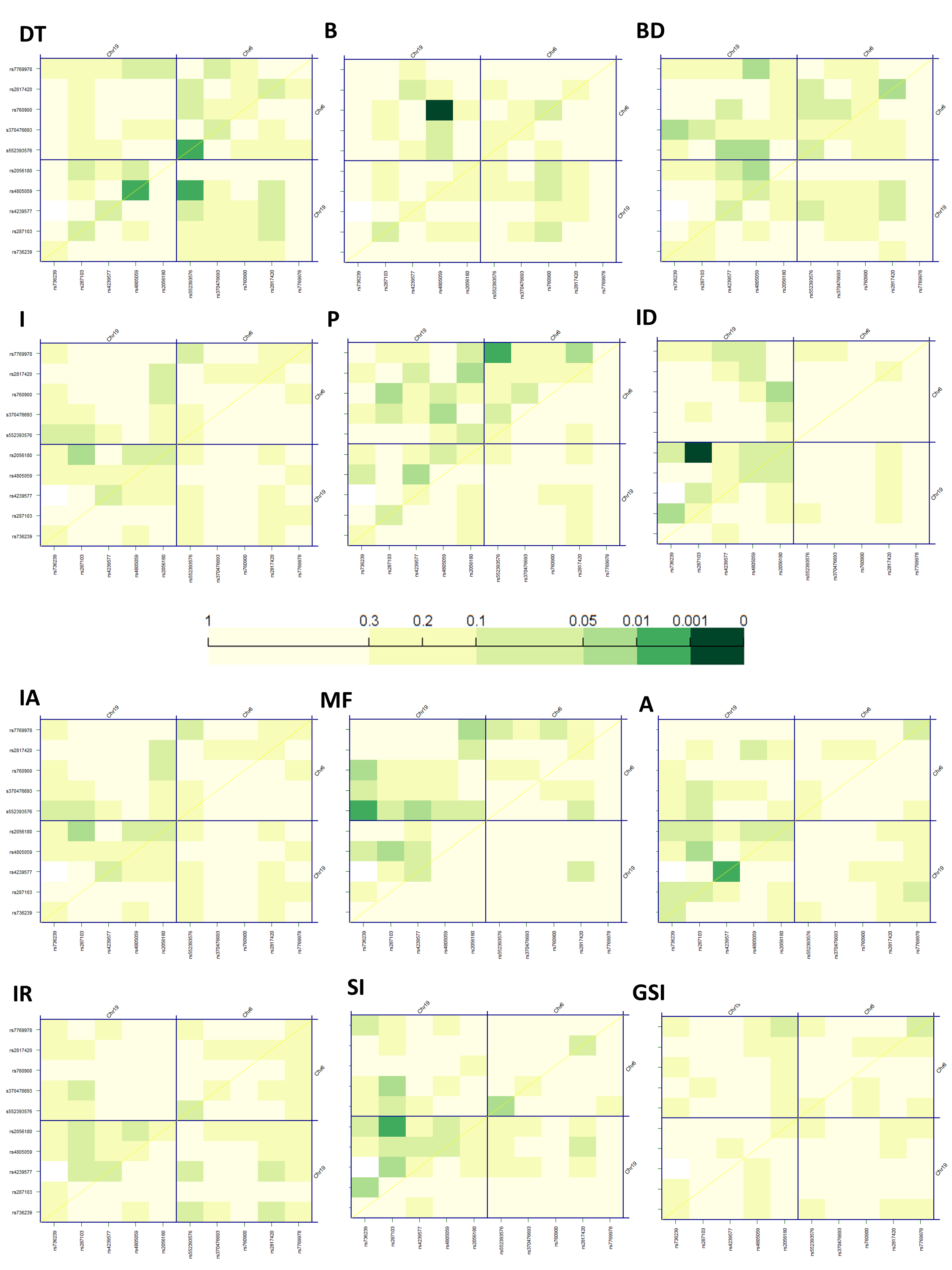

Supplement: Supplementary file 2 [file BRB3-7-e00784-s002.TIF]

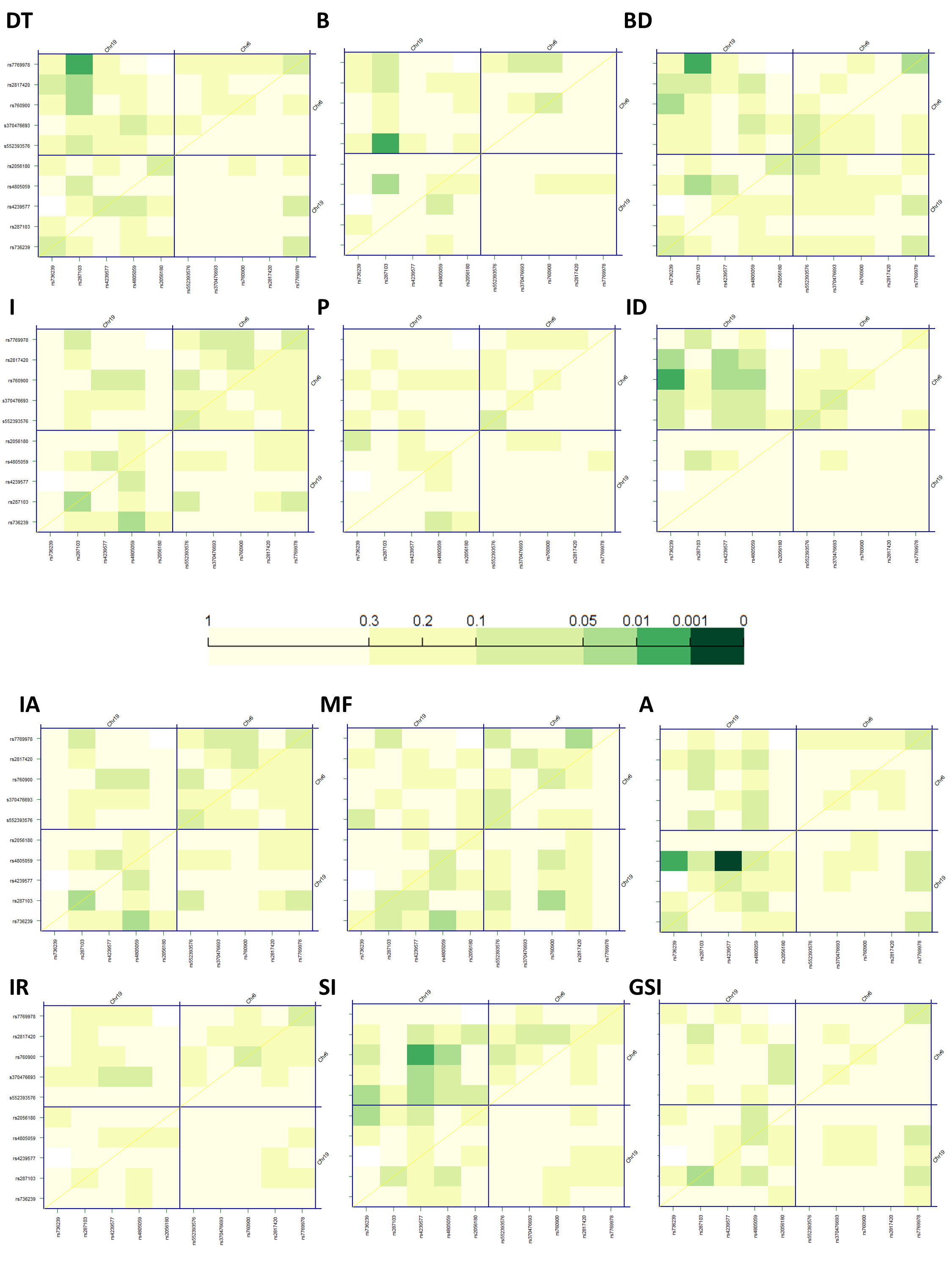

Supplement: Supplementary file 3 [file BRB3-7-e00784-s003.TIF]
