# Supplementary material for: Influence of TFAP2B and KCTD15 genetic variability on personality dimensions in anorexia and bulimia nervosa
Source: Brain Behav. 2017 Jul 27;7(9):e00784. doi: 10.1002/brb3.784 (PMC5607548; doi:10.1002/brb3.784)
Supplement: Supplementary file 4 [file BRB3-7-e00784-s004.docx]

**Supplementary Table S1.** Frequency of haplotypes identified in patients with eating disorders and obesity and in control subjects

|  |  |  |  |  |  | TOTAL ED | AN | BN | Obese^§^ | Controls^§^ |
| --- | --- | --- | --- | --- | --- | --- | --- | --- | --- | --- |
| *TFAP2B* | rs760900 | rs2817420 | rs7769978 | **^ɸ^**rs370476693 | rs552393576 |  |  |  |  |  |
| **1* | T | C | T | L | A | 0.387 | 0.340 | 0.465 | - | - |
| **2* | C | T | T | S | G | 0.147 | 0.168***** | 0.111 | - | - |
| **3* | C | C | T | S | A | 0.143 | 0.160 | 0.115 | - | - |
| **4* | C | C | C | S | A | 0.127 | 0.142 | 0.103 | - | - |
| **5* | C | C | T | L | A | 0.121 | 0.112 | 0.139 | - | - |
| *KCTD15* | rs736239 | rs287103 | rs4239577 | rs4805059 | rs2056180 |  |  |  |  |  |
| **1* | C | T | C | G | T | 0.370 | 0.359 | 0.389 | 0.327 | 0.304 |
| **2* | G | C | T | A | T | 0.215 | 0.219 | 0.206 | 0.287 | 0.209 |
| **3* | C | C | C | A | T | 0.149 | 0.139 | 0.167 | 0.151 | 0.168 |
| **4* | C | C | C | G | T | 0.109 | 0.102 | 0.119 | 0.096 | 0.085 |
| **5* | G | C | C | G | T | 0.087 | 0.115***** | 0.040 | 0.080 | 0.082 |
| **6* | G | C | T | A | C | 0.072 | 0.067 | 0.079 | 0.060 | 0.137**^§^** |

*p < 0.05 vs. BN patients; ^§^p<0.05 vs. obese, AN and BN patients;

**^§^**Only the three first SNPs of *TFAP2B* were analyzed in the obese and control groups.

**^ɸ^**L, long allele (five repeats); S, short allele (four repeats).

**Supplementary Table S2**

Distribution of rs2817420 between obese, anorexia and bulimia patients. Percentages of carriers are shown for each genotype.

|  | Obese | Controls | p-value* | AN | p-value* | BN | p-value* |
| --- | --- | --- | --- | --- | --- | --- | --- |
| *Codominant* |  |  |  |  |  |  |  |
| *C/C* | 54.7 | 70.1 | 0.062 | 65.4 | 0.049 | 79.4 | 0.008 |
| *C/T* | 41.3 | 26.6 |  | 31.7 |  | 19 |  |
| *T/T* | 4.0 | 3.4 |  | 2.9 |  | 1.6 |  |
| *Dominant* |  |  |  |  |  |  |  |
| *C/C* | 54.7 | 70.1 | 0.020 | 65.4 | 0.024 | 79.4 | 0.002 |
| *C/T-T/T* | 45.3 | 29.9 |  | 34.6 |  | 20.6 |  |
| *Recessive* |  |  |  |  |  |  |  |
| *C/C-C/T* | 96.0 | 73.4 | 0.813 | 97.1 | 0.131 | 98.4 | 0.386 |
| *T/T* | 4.0 | 26.6 |  | 2.9 |  | 1.6 |  |

*p-values vs. obese individuals.
